# The Influence of Disorder in the Synthesis, Characterization and Applications of a Modifiable Two-Dimensional Covalent Organic Framework

**DOI:** 10.3390/ma14010071

**Published:** 2020-12-25

**Authors:** Jordan Brophy, Kyle Summerfield, Jiashi Yin, Jon Kephart, Joshua T. Stecher, Jeramie Adams, Takashi Yanase, Jason Brant, Katie Dongmei Li-Oakey, John O. Hoberg, Bruce A. Parkinson

**Affiliations:** 1 Department of Chemistry, University of Wyoming, Laramie, WY 82071, USA; jordanfbrophy@gmail.com (J.B.); jkephart@uw.edu (J.K.); Joshua.stecher@gmail.com (J.T.S.); jbrant270@yahoo.com (J.B.); 2 School of Energy Resources, University of Wyoming, Laramie, WY 82071, USA; ksummerf@uwyo.edu; 3 Department of Chemical Engineering, University of Wyoming, Laramie, WY 82071, USA; yinjiashi@gmail.com (J.Y.); dli1@uwyo.edu (K.D.L.-O.); 4 Western Research Institute, University of Wyoming, Laramie, WY 82071, USA; Jeramie.adams@uwyo.edu; 5 Division of Applied Chemistry, Hokkaido University, Sapporo 060-0808, Japan; yanase42@eng.hokudai.ac.jp

**Keywords:** nanoporous covalent organic frameworks, carbonylation, ion sieving, disorder, carboxylated pores

## Abstract

Two-dimensional covalent organic frameworks (2D-COFs) have been of increasing interest in the past decade due to their porous structures that ideally can be highly ordered. One of the most common routes to these polymers relies on Schiff-base chemistry, i.e., the condensation reaction between a carbonyl and an amine. In this report, we elaborate on the condensation of 3,6-dibromobenzene-1,2,4,5-tetraamine with hexaketocyclohexane (HKH) and the subsequent carbonylation of the resulting COF, along with the possibility that the condensation reaction on HKH can result in a trans configuration resulting in the formation of a disordered 2D-COF. This strategy enables modification of COFs via bromine substitution reactions to place functional groups within the pores of the materials. Ion-sieving measurements using membranes from this COF, reaction of small molecules with unreacted keto groups along with modeling studies indicate disorder in the COF polymerization process. We also present a Monte Carlo simulation that demonstrates the influence of even small amounts of disorder upon both the 2D and 3D structure of the resulting COF.

## 1. Introduction

Two-dimensional covalent organic frameworks (2D-COFs) are an emerging class of polymeric materials due to their expansive range of desirable properties [1,2,3,4]. 2D-COFs have regular porous structures that can be designed by the choice of monomers used in the polymerization reaction [2,5,6,7]. The bottom-up approach used in the synthesis of 2D-COFs relies on the extensive tools of synthetic organic chemistry that provide extraordinary control of both pore size, shape and spacing based on the choice of synthetic monomers [8]. Highly ordered materials can then be exploited in applications such as membrane separations, optoelectronics and energy storage [9]. There have been a commonly used set of polymerization reactions employed to produce most 2D-COFs, most notably borate chemistry and condensation reactions between ketones and amines [10,11]. A particularly noteworthy example is nitrogen containing g-C_2_N, a hexagonal network of nitrogen-lined small pores produced by a condensation reaction between hexaketo cyclohexane (HKH) and benzene hexamine termed “holey graphene” [12]. This very stable material has a highly delocalized aromatic backbone and is reported to be highly ordered and have useful semiconductor properties based on high carrier mobilities measured in field effect transistor (FET) devices. Although this material is very interesting, it is not amenable to modifying its basic pore structure, such as the strategy we recently reported [13,14]. Recent reports have replaced the hexaamino benzene with 1,2,4,5-tetraamino benzene illustrating that COFs can be formed from this two-fold symmetric monomer [15,16,17,18]. The focus of this paper is to report the reaction of 3,6-dibromo-1,2,4,5-tetraamino benzene with HKH, which enables the functionalization of the COF pores either post or pre-COF formation allowing for the synthesis of materials for specific applications [18,19]. We also visualize the occurrence of disorder in the materials via a Monte Carlo simulation of the expected growth mechanism of 2D-COFs. This disorder can occur when the tetraamine adds to the pair of ketone groups on HKH across from, rather than adjacent to, where the first tetramaine added produces what we refer to as a linear defect or disorder. The simulation provides a conceptional image into the disorder in the structure of the final products and how the presence of the linear defects might affect its performance in a given application.

## 2. Materials and Methods

**General.** All reagents were ACS grade and were used without further purification. ACS glacial acetic acid and anhydrous *N*-Methyl Pyrrolidone (NMP) were used as received; DMF was purified before use. ^1^H and ^13^C NMR spectra were recorded with deuterated solvents using Bruker 400 or 600 MHz NMR spectrometers, calibrated using the stated residual protonated solvent as an internal reference. Chemical shifts (δ) are reported in parts per million (ppm) and coupling constants (*J.*) are measures in hertz (Hz). Fourier-transform infrared (FT-IR) spectroscopy was performed with a Perkin Elmer Spectrum One spectrometer.

**Synthesis of (2) via Hydrogenation.** A Parr reactor was purged with argon then loaded with **3** (384 mg, 1.00 mmol), 10% Pd(OH)_2_/C (10 mg) and EtOH (10 mL). The reactor was pressurized to 120 psi with H_2_ and heated at 100 °C in an oil bath for 40 h. After cooling to room temperature (RT), the solution was vacuum filtered through celite with hot EtOH and the filtrate concentrated under vacuum to give a brown solid (293 mg, 99% yield), which was placed under argon and used immediately. ^13^C NMR (100 MHz, DMSO-d_6_; ppm): 143.1, 96.0. IR (cm^−1^): 3136, 1546.

**Synthesis of COF 4.** To a round-bottom flask equipped with stir bar and condenser were added **3** (1.07 g, 2.79 mmol), Zinc (2.70 g, 41.28 mmol), AcOH (30 mL) and 3 drops of H_2_O. The mixture was heated with stirring at 60 °C for 6 h, cooled and filtered, washing the solids with AcOH. The AcOH was removed under vacuum and the solid purged with argon. NMP (5 mL), hexaketocyclohexane 8 H_2_O (2.79 mmol) and 3 drops of H_2_SO_4_ were added and the mixture was heated at 175 °C for 24 h. The mixture was cooled to RT and the black solid was crashed out with ether. Soxhlet extraction with H_2_O (12 h) then EtOH (12 h) followed by drying under high vacuum for 3 days gave 604 mg of black solid. Alternatively, **2** from the hydrogenation procedure above was used in replace at equal molar equivalent to **1**.

**Synthesis of COF 5.** To a glass Parr reactor was added **4** (400 mg), K_2_CO_3_ (335 mg, 2.40 mmol), PdCl_2_(PPh_3_)_2_ (65 mg, 0.09 mmol), DMF (5 mL) and H_2_O (2 mL). The mixture was purged 3× with CO and then pressurized to 50 psi followed by heating with stirring at 50 °C for days. The mixture was cooled to RT, filtered, Soxhlet extracted with H_2_O (22 h) then EtOH (9 h) followed by drying under high vacuum for 3 days to give 300 mg of a black solid.

**Characterization methods.** An amount of 1.25 mg of COF **5** was initially dispersed in 20 mL of 10 mM NaOH solution to convert the group to COONa by sonicating the mixture for 24 h. The COO- ions in the solution were then analyzed with 0.020021 M HCl solution (standardized by tris(hydroxymethyl) aminomethane, 99.9%, Sigma). The amount of COOH groups in the COF samples were calculated from the consumption of the standardized HCl solution, which equals to VHCl × CHCl, where VHCl is the titrant volume (mL) and CHCl is the molar concentration of the titrant, respectively. Powder X-ray diffraction (XRD) was carried out using a Rigaku Smart Lab X-ray diffractometer (XRD, with Cu Kα radiation source (λ = 1.544 A). Using peak positions, d-spacing was calculated based on Bragg’s law using peak positions. The morphology of COF films was analyzed by SEM (FEI Quanta FEG 450 FESEM. All the samples were coated with carbon. For cross-sectional imaging, the films were immersed in liquid nitrogen and were then snapped with flat tweezers. TEM was performed using a FEI Tecnai G2 F20 200 kV TEM. TEM grids of the as-prepared samples were prepared by drop-casting the dispersed COF solution onto holey carbon film TEM grids and then wicking away excess solution after several seconds. Thermal gravimetric analyses (TGA) were performed in a TA Instruments SDT Q600 thermogravimetric analyzer for temperatures ranging from 25 to 1200 °C, with a heating rate of 3 °C/min under air and argon, respectively (Figure S9). 

**Membrane fabrication.** Graphene oxide (GO) films were prepared by conventional doctor blade casting of a viscous GO ink (~40 mg/mL) onto a track-etched polycarbonate membrane (TEPC, pore size: 0.2 μm). Resulting hybrid films were dried overnight under ambient lab conditions (Akbari et al., 2017). Using the same solution preparation procedure as for titration tests, COF **5** was sufficiently exfoliated in basic water (10 mM NaOH) by ultra-sonication for 24 h. Sequentially, COF **5** films were fabricated by drop-casting the dispersed COF **5** solution onto a TEPC support followed by drying overnight in a vacuum oven at 60 °C. 

**Cation selectivity.** The apparatus for ion selectivity measurement consists of a U-shaped tube with a diameter of 1.6 cm, which was divided by a membrane into two compartments, referred to as feed and permeate side, respectively. The feed compartment was filled with 0.1 M of different ammonium solutes in ethanol. The permeate side was filled with pure ethanol. Both sides were filled to a constant level of 10 cm. Each side has a platinum electrode that is connected to a potentiostat (Ivium Technologies). Ion transport can be probed by monitoring electrical current of the whole setup under a wide voltage range (0–2 V). Ethanol was chosen as the electrolyte since COF **5** membrane dissolves in water, possibly due to the -COOH groups. 

The cation selectivity of COF **5** and GO membranes were examined by evaluating their conductivity towards various organic ammonium salts, with anion being the same (p-toluenesulfonate, pTSO). The radii and hydrated radii of the organic ammonium cations used are summarized in Table S1 of the SI.

## 3. Results

**Figure 1:**
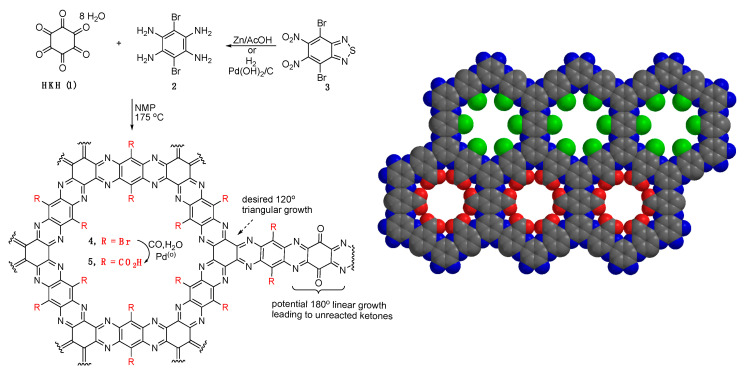
Left: synthetic routes for the synthesis of covalent organic frameworks (COFs) **4** and **5**, with a possible linear defect. Right: space-filling model with bromines in green, top and CO_2_H moieties in red, bottom.

**Synthesis of COF 4 and 5**. Synthesis of a bromine containing COF involved condensation of hexaketocyclohexane (HKH) **1** [19,20] with dibromotetraamine **2** using conditions reported by Baek and co-workers (Figure 1) [12]. Dibromo tetraamine **2** is typically prepared from benzothiadiazole **3** [21,22] by zinc reduction in acetic acid and used without isolation by filtration and subsequent reaction in the acetic acid [21,23,24]. We found that the use of the higher boiling *N*-Methyl Pyrrolidone (NMP) as a solvent was necessary and thus the acetic acid was removed in-vacuum after filtration of **2**. Condensation of **1** and **2** in NMP with trace H_2_SO_4_ at 175 °C for 24 h produces a black solid. Characterization of **4** indicated a dependence on both solvent and temperature of the condensation reaction as noted in the supplementary information. For example, lower temperatures gave rise to amorphous materials. We also developed an alternative reduction in **3** using hydrogenation over Pd(OH)_2_/C in ethanol that produces tetraamine **2** as a brown solid in 99% yield. We found this to be a significantly easier method for the preparation of **2**; however, as the free-base it had to be used immediately otherwise inferior samples of COF **4** were produced. Alternatively, the use of **2** from the zinc reduction consistently produced quality COF as determined by TEM studies (see Figure S8 in the Supplementary Materials for a comparison of TEM images). 

Two methods for post-synthetic modification were studied. The incorporation of bromines into the pores of **4** provides for a convenient method for metal-catalyzed substitutions. Thus, transformation of COF **4** into the carboxylic acid **5** was performed next. COF **4** was treated with PdCl_2_(PPh_3_)_2_, K_2_CO_3_, 1 atm CO, in H_2_O/DMF at 90 °C for three days. Upon filtration and Soxhlet extraction of the black solid, the IR displayed a C=O stretch at 1680 cm^−1^ and a broad OH stretch from 3300 to 3000 cm^−1^ both consistent with carboxylic acid COF **5** (see Supplementary Figure S4 for comparison of **4** and **5**). 

**Figure 2:**
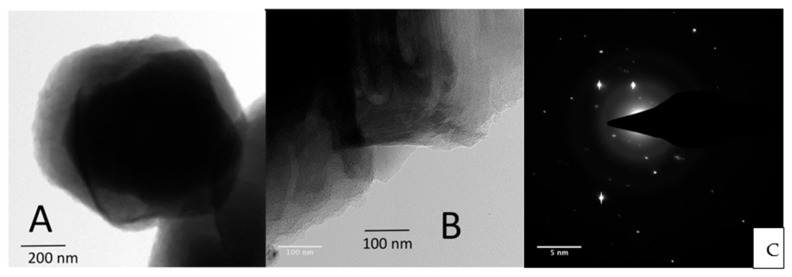
(**A**) and (**B**): HRTEM micrographs of brominated COF **4**, (**C**): diffraction of COF **4**. Enlarged images of A and B are included in the Supplementary Materials as Figures S7 and S8.

Additional characterization of both **4** and **5** further established the structures. TEM images were obtained for COF **4** as higher contrast images are more easily obtained on the brominated structure due to the presence of the highly electron scattering bromine atoms (Figure 2). We were able to locate regions that did show crystallites with stacked near hexagonal nanocrystallites (Figure 2A) as expected given the hexagonal symmetry of the COF. Higher magnification TEM images as shown in Figure 2B show lattice periodicity on the periphery of the flakes. The periodicity of these linear and hexagonal structures ranges from about 3.2 to more than 5 nm, which was larger than the pore spacing measured from the molecular modeling of 1.66 nm of the single layer structure. Since these distances are observed on areas where there are multiple COF layers, they are probably due to Moiré patterns from selected areas of multilayers each with some order. Electron diffraction of the COF flakes illustrated an inconsistent hexagonal pattern as was more routinely observed in TEM images of COFs from our previously published work.^13^ Rather more random diffraction spots or diffraction rings were measured that are typical of either less ordered or amorphous materials (Figure 2C). The powder X-ray diffraction results of COFs **4** and **5** reveals a main broad peak at 2θ = 24°, which is attributed to the stacking arising from the (002) plane (see Supplementary Figure S5), corresponding to interlayer d-spacing of about 0.37 nm. The width of this peak indicates the COF is semi-crystalline but not highly ordered.

**Figure 3:**
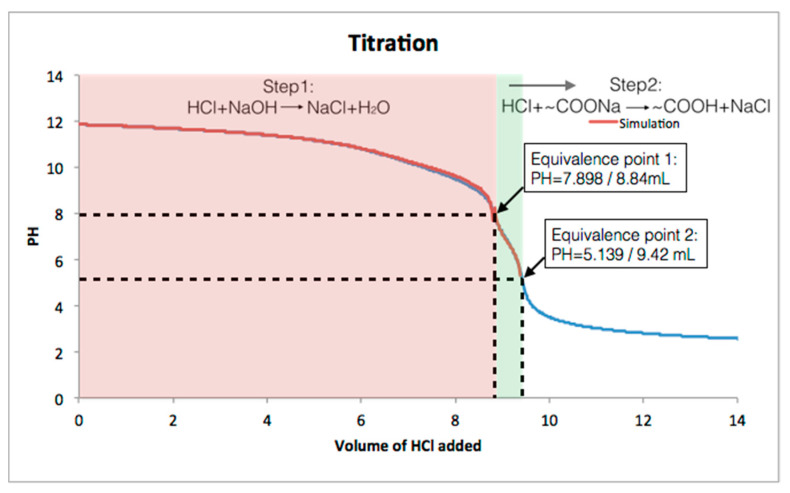
Titration curves for suspensions of COF **5**. Red curve is the simulation and blue curve is the data.

Next, we carried out acid-base titration experiments to measure the amount of -COOH groups in the carboxylated COF samples. COF **5** was dispersed in a 10 mM aqueous NaOH solution and sequentially titrated by standardized 20.02 mM HCl solution. The titration assumed two reaction steps as shown in the Figure 3, where HCl first neutralized the excess NaOH, followed by the protonation of -COONa in the COF. Figure 3 shows that simulated titration curves matched the experimental titration curve as expected. The following calculation was based on the two reactions. The amount of the COOH groups the sample of COF was calculated to be 11.6 × 10^−6^ moles (see Experimental Section for detail). However, the theoretical amount of COOH in 1.25 mg of COF is 9.77 × 10^−6^ moles indicating that the experimental COOH amount is ~16% higher than the theoretical amount. This result indicates that the synthesized COF is not perfectly ordered, due to potential linear defects in its structure as shown in Figure 1. The fact that the experimental result is higher may be due to the fact that this calculation requires both the molecular weight of the COF be known as well as the particle size. The molecular weight of the ordered COF is precise however since the disorder can result in both open space and extra functional groups in the pores from defects above or below a given layer this results in an unknown molecular weight and thus number of actual carboxylates in the structure is also not precise. The termination of the edges of COF flakes by either amine or ketone moieties will also influence both the measurement of free ketone groups and carboxylates (in COF **5**), however, this effect is not significant with flake sizes of about 100 nm or greater since a 100 nm 2D-COF flake has at most about 6% terminating groups that could be divided between amines and ketones. 

The second post-modification reaction was performed by converting unreacted COF carbonyls to imines with the use of a primary amine. To demonstrate this, a sample of COF **4** was placed in an NMR tube loaded with DMSO-d_6_, isopropyl amine, cyclohexane and sealed under argon. The mixture was heated to 100 °C and the disappearance of the amine was monitored by ^1^H NMR using cyclohexane as a standard. In multiple experiments, the disappearance of the amine was observed in all cases along with the appearance of a broad peak shifted 0.1 ppm downfield of the amine methyl group (see Supplementary Figure S6). Filtration of the COF through silica followed by ^1^H NMR of the filtrate displayed only the cyclohexane standard, thus verifying the incorporation of the isopropyl amine into the COF. The molar amount of isopropyl amine reacted corresponds less than the number of ketone groups expected with the possible termination of all 2D COF flakes with unreacted keto groups even if it had no defects due to the existence of the linear defects in Figure 1. Most importantly, this experiment demonstrates a facile method for post COF synthesis to modify edge sites in a COF sample and may be useful to stabilize the edges, passivate defects or even cross-link smaller COF flakes into larger aggregates using branched diamines.

**Figure 4:**
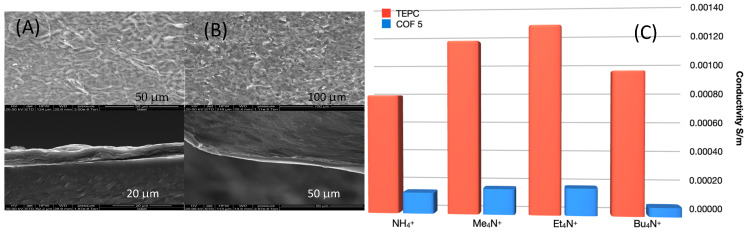
SEM images of (**A**) graphene oxide (GO) and (**B**) COF **5** membranes. Top row: top surface; bottom row: cross-section. (**C**): Cation selectivity of neat track-etched polycarbonate (TEPC), GO (GO membrane had less than 0.0000 × S/m) and COF/TEPC hybrid membranes.

**Ion-sieving measurements**. Given the small pores of these 2D-COFs, we next used our previously published ion-sieving method to investigate the ion conductivity of a COF **5** membrane [13]. This work demonstrated that a more highly ordered 2D-COF with carboxylated pores, synthesized using precursors that eliminated the possibility of the linear defects in their structure, rejects tetraalkyl ammonium cations of a size larger than the COF pore diameter. In that case R_4_N^+^ ions (R = linear alkyl chains of various lengths) that are smaller than the COF pore diameter passed through that COF membrane and provided a measurement of ion conductivity based on pore size, i.e., smaller sized ions pass through an ordered COF membrane whereas larger ones are almost completely rejected. In this study, COF **5** and GO hybrid membranes were made by vacuum filtration using track-etched polycarbonate (TEPC) as a membrane support. GO membranes were used for comparison given their proposed use as size-selective ion transport membranes [25]. Figure 4A,B illustrates the top surface SEM images of the COF membranes that show a similar morphology to GO membranes. While GO cross-sectional images display the well-known layered structure, COF cross-sections presented a continuous, dense layer on top of the TEPC substrate. 

Figure 4C illustrates the results from the conductivity measurements. As expected, GO membranes show nearly no ion conductivity as ion transport can occur only via the tortuous path in the thin spaces between the layers that are much smaller than the ions used in this study. Moreover, the TEPC control possess the expected high ion conductivity due to its pore size being much larger than the ions. The COF**5**/TEPC-supported membrane showed the lowest conductivity for Bu_4_N^+^ compared to the other smaller tetraalkyl ammonium ions. However, the Bu_4_N^+^ ion has ionic radii of more than twice that of COF **5′**s pore diameter suggesting that disorder in COF **5** can result in a pore structure with larger pores as can be seen in the simulations presented below, which was also confirmed with Brunauer-Emmett-Teller (BET) analysis (Supplementary Figure S10). The lower conductivity of NH_4_^+^ compared to Me_4_N^+^ and Et_4_N^+^ in both the control TEPC and COF membrane may be attributed to the fact that the NH_4_^+^ is prone to form hydrogen bonds to hydrophilic groups, resulting in larger ion radius in ethanol than its intrinsic radius. R_4_N^+^ ions (R = alkyl) become less hydrophilic as the chain length of R increases, causing the ionic radius to be closer to their intrinsic one. The trade-off between the increased ion size and decreased hydrophilicity likely explains the slightly higher conductivity in Et_4_N^+^ than Me_4_N^+^_._ Even so, COF **5** does illustrate the ability to inhibit transfer of ions with a specific size threshold.

**Growth Mechanism Simulation.** The condensation reaction between HKH **1** and tetraamine **2** can result in either the preferred reaction of three tetra-amines around one HKH (120° triangular growth) or a linear 180° condensation leaving two unreacted ketone groups, as illustrated in Figure 1. It is often presumed that since these Schiff base type condensation reactions are reversible, the COF forming condensation reactions will reach an equilibrium where highly ordered materials are the thermodynamic product and will eventually form. However, equilibrium in this case can be difficult to achieve due to the multiple imine bonds formed in these double condensation reactions making dissociation reactions more unlikely due to a chelate effect. This leads to uncorrected errors that, even if they are as low as one percent, this small level can affect the structure and perhaps the performance of the final materials. Disorder in other 2D-COFs has been noted as Nguyen and Grunwald reported on the origins of disorder in the formation of boronate ester COFs that can be formed closer to equilibrium conditions than these double condensation reactions [26]. Furthermore, the issues in reversibility and crystallinity in COFs have recently been reviewed that highlight the issues with engineering error-free COFs [27].

Accordingly, we wanted to develop a convenient method for the visualization of this possibility as it is worthwhile to remember that, unlike most organic synthetic reactions where a crystalline product is formed from nucleation and growth of a crystal from small molecules, formation of highly crystalline 2D-COFs is highly dependent on the initial nucleation since small nuclei are not consumed to promote the growth of larger crystallites as in the Ostwald ripening process. The covalent bonding of COF growth can also result in small nuclei aggregating in random ways via crosslinking reactions. These aggregates may promote further disorder by not stacking properly into ordered multilayers. Additionally, aggregation of small flakes, and the linear errors discussed above, could result in “orphan bonds” as discussed below that may be either amine or keto terminated and thus not accessible to larger monomers or small aggregates to continue growth.

Thus, to better visualize the growth mechanism of these 2D COFs and visualize the 2D and 3D COF structures, we developed a Monte Carlo simulation to help envision the effects the linear addition of the tetra amine on the final 2D and 3D structure of the polymer. Included, and equally important, is the influence of grain boundaries on the growth of even linear defect-free platelets. The simulation allows for controlling the probability of forming the linear structure vs. the desired triangular growth around the core of HKH as the various possible unreacted sites are interrogated and subsequent reaction probabilities are applied. It also allows for multiple nucleation sites that would occur if two small growing platelets come together shortly after nucleation and then, if the growing lattices are out of phase, form grain boundaries either in a linear error-free fashion or with incorporation of the linear defects. The simulation starts with a lattice of symmetry related points, including the midpoint of the pore that would be occupied only when linear defects occur. The rules of the simulation are that when a 2D propagating structure encounters a point where there is no possibility of connecting to an unreacted diketone site on the same layer, it has a 50% probability of continuing to grow on a layer either above or below that layer. If that layer is already blocked by growth, it will have a 100% probability of continued growth on the unblocked layer. There is also a possibility of “orphan bonds” where there is no possibility of propagating on either above or below layers. These orphan bonds are either single or multiple depending on whether further growth is blocked on that layer in only one or both directions. This growth mechanism is related to the growth mechanism of other 2D solid-state materials where crystals thicker than the monolayer are usually formed by so-called screw dislocations since edge growth is favored over nucleation and growth of a new layer on a van der Waals surface. The simulation also allows for simulating up to 15 layers above and below the nucleation plane to allow examination of the propagation of these defects throughout a more 3D platelet. The simulation is available online for readers to experiment showing the implications on the final structure of the materials based on the probability of forming the linear defects (https://chemicallattice.firebaseapp.com). Parameters such as the statistical number of linear defects, the size of the lattice in both 2D and 3D and the number of nucleation sites can be varied to see their influence on the final structure. The simulation also assumes that the pores are aligned between the layers. We have some evidence in these materials from the TEM images that this is true on both Br substituted and carboxylated pores due to the van der Waals forces between bromines and hydrogen bonding interactions between carboxyl groups controlling the layer stacking.

**Figure 5:**
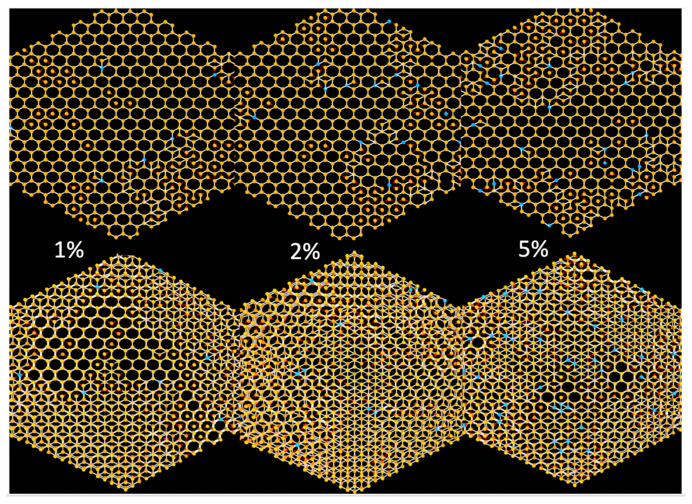
Screen shots of the Monte Carlo simulation of a 9-layer COF simulation that illustrates the influence of the percentage of linear defects (blue lines are not terminated in a hexagon and indicate links to a layer above or below) on the lattice. The top row shows the single layer where growth was initiated (nucleation layer), while the bottom row shows the central three layers, with one above and one below the nucleation layer. Orphan bonds are indicated by small red rods. The 1% defect probability simulation shows that a high percentage of hexagonal channels can span multiple layers but will not line up to form channels even with a few more than 3 layers. Remarkably, a mere 2–3% of linear defects will seriously affect the ability of the layers to form pores spanning the width of the three-layer slice of the nine-layer simulation.

The top row of Figure 5 shows images of single layer slices generated by the simulation with low probabilities (1, 2 and 5%, respectively) of producing the linear defects as well as the bordering layers above and below this layer in the bottom half of the figure. The linear defects produce multilayer lattices, via the screw dislocation-like growth mechanism, that will eventually have many of these lattice points not on the same plane when viewed down the c-axis of the structure. The simulation also allows for increasing the layer separation so that you can easily see all of the interlayer connections when rotating the original view. This program does not verify or establish disorder in a COF but conveniently allows researchers to see the effects prior to selecting COF reactants.

## 4. Conclusions

We have presented an experimental approach that allows for the incorporation of bromines into a small pore 2D-COF that is able to undergo subsequent functional group transformation. Membranes fabricated from carboxylated COF **5** show ion conductivity for various sized quaternary ammonium ions. Well-ordered regions in the TEM images did confirm the structure of the COFs, but pXRD indicates some disorder. Furthermore, we have illustrated two simple post-COF modifications on the COF that enables the incorporation of additional functionality. These modifications also hold potential for cross-linking smaller COF flakes into larger aggregates. Finally, we have developed a simple computational simulation that allows for the visualization of potential error in COF growth.

## Data Availability

Data is contained within the article or Supplementary Material.

## Supplementary Materials

The following are available online at https://www.mdpi.com/1996-1944/14/1/71/s1, Figure S1: ^13^C NMR of 3 in DMSO-d6 for comparison to 2 (below in Figure S2), Figure S2: ^13^C NMR of 2 in DMSO-d6, Figure S3: IR of 2, Figure S4: IR of COF 4 (top) & 5 (bottom), Figure S5: PXRD of COF 4 top and COF 5 bottom, Figure S6: ^1^H NMR experiment showing the incorporation of iPrNH_2_ into COF 4. A 20.5 mg sample COF 4 (equal to 0.154 mmol HKH and 0.154 mmol 2) was reacted with isopropyl amine (18ul, 0.22 μmol). Spectrum (a) is time zero. Spectrum (b) is after 6 hours and (c) is 12 h in which all the CH_3_ signals at 0.95 have disappeared. After filtration of the solid COF, the new peak at 1.05 disappears. Figure S7: Enlarged TEM image of COF 4 from Figure 2 of manuscript, Figure S8: Enlarged TEM image of COF 4 from Figure 2 of manuscript (top); and TEM image of COF 4 made from tetraamine 2 synthesized by reduction of 3 using hydrogenation over Pd(OH)_2_/C in ethanol, Figure S9: Thermogravimetric analysis and differential scanning calorimetry on COF 5 under (a) air and (b) argon. TGA/DSC analysis obtained from 25 °C to 1200 °C at a heating rate of 3 °C min^−1^ of the material under both argon and air shows a loss of mass at low temperatures indicating absorbed or adsorbed solvent or water also providing additional mass without carboxyl moieties. TGA/DSC curves under air showed the COF was completely oxidized at 600 °C. TGA/DSC under argon atmosphere shows that the COF 5 is stable up to 400 °C with ~30% weight loss and further gradual weight loss past 900 °C. Decarboxylation maybe responsible for the initial weight loss, Figure S10: BET Surface area plot (top) and pore size distribution of COF 5. Table S1: Radii and hydrated radii of R_4_N^+^ cations.
