# Supplementary material for: The Influence of Disorder in the Synthesis, Characterization and Applications of a Modifiable Two-Dimensional Covalent Organic Framework
Source: Materials (Basel). 2020 Dec 25;14(1):71. doi: 10.3390/ma14010071 (PMC7795170; doi:10.3390/ma14010071)
Supplement: Supplementary file 1 [file materials-14-00071-s001.pdf]

Supporting Information

The influence of disorder in the synthesis, characterization and applications of a modifiable two-dimensional Covalent Organic Framework

Jordan Brophy,[a] Kyle Summerfield,[b] Jiashi Yin,[c] Jon Kephart,[a] Joshua T. Stecher,[a] Jeramie Adams,[d] Takashi Yanase,[e] Jason Brant,[a] Katie Dongmei Li-Oakey,[c] John O. Hoberg[a*] and Bruce A. Parkinson[a,b*]

[a] Department of Chemistry, University of Wyoming

[b] School of Energy Resources, University of Wyoming

[c] Department of Chemical Engineering, University of Wyoming

[d] Western Research Institute, University of Wyoming

[e] Division of Applied Chemistry, Faculty of Engineering, Hokkaido University

Contents

	Page:
On-line simulation	3
Polymerization reactions	3
Figure S1. ^{13}C NMR of **3**	4
Figure S2. ^{13}C NMR of **2**	5
Figure S3. IR of **2**	6
Figure S4. IR of COFs **4 & 5**	7
Figure S5. PXRD of COFs **4 & 5**	8
Figure S6. Isopropyl amine incorporation into COF **4**	9
Figure S7 & 8. TEM images of COF **4**	10-11
Figure S9. DSC & Thermogravimetric analysis	12
Table 1. Radii and hydrated radii of R_4N^+ cations.	12
Figure S10. BET analysis of COF **5**	13

Directions for use of the on-line simulation https://chemicallattice.firebaseapp.com:

You are initially shown a rhombic grid of hexagonal lattice points representing the hexagonal lattice of the COF and a point in the center of the ordered COF that gets occupied when there are linear errors on the lattice. The sliders on the upper left allow one to change the lattice grid size up to a rhombus of 35 symmetry points on a side, the distance between the layers, to better view the interlayer links the number of layers in the simulation and the number of layers shown on the screen (visible layers). You might want to have outer layers not shown to make the simulation less dense but to provide for the accurate simulation of those layers to include growth down from layers above and up from layers below the viewed layers. The regenerate button initializes a simulation by erasing the previous simulation. The linear chance slider in the lower left allows the adjustment of the probability of a linear error in the 2D COF. The speed slider adjusts the rate that the simulation proceeds to enable viewing the formation of the structure. The pitch and yaw adjustments allow for changing the view of the simulation from top to side views. A single or multiple nucleation sites can be generated by **clicking on one or many lattice points in the rhombic grid**. After completion of the simulation the number of orphan bonds, layer connection links and empty lattice points is shown in tables on the right for all the layers used in the simulation visible or not.

COF alternative conditions that were tested:

Table 1.

No.	Solvent	Temp./°C	Rx time (hours)
1	1 mL 3 M CH3COOH & 1 mL ethylene glycol	120	64
2	2 mL NMP & 50 μL 3 M H2SO4	120	19
3	1 mL 3 M CH3COOH & 1 mL ethylene glycol	120	19
4	1 mL 3 M CH3COOH & 1 mL ethylene glycol	120	24
5	2 mL NMP & 50 μL 3 M H2SO4	120	24
6	2 mL NMP & 50 μL 3 M CH3COOH	120	24
7	1 mL NMP & 1 mL 3 M CH3COOH	120	24
8	2 mL NMP 50 μL 3 M H2SO4	150	24
9	2 mL NMP & 50 μL 3 M CH3COOH	150	24
10	5 mL NMP & ~200 μL conc. H2SO4	175	24

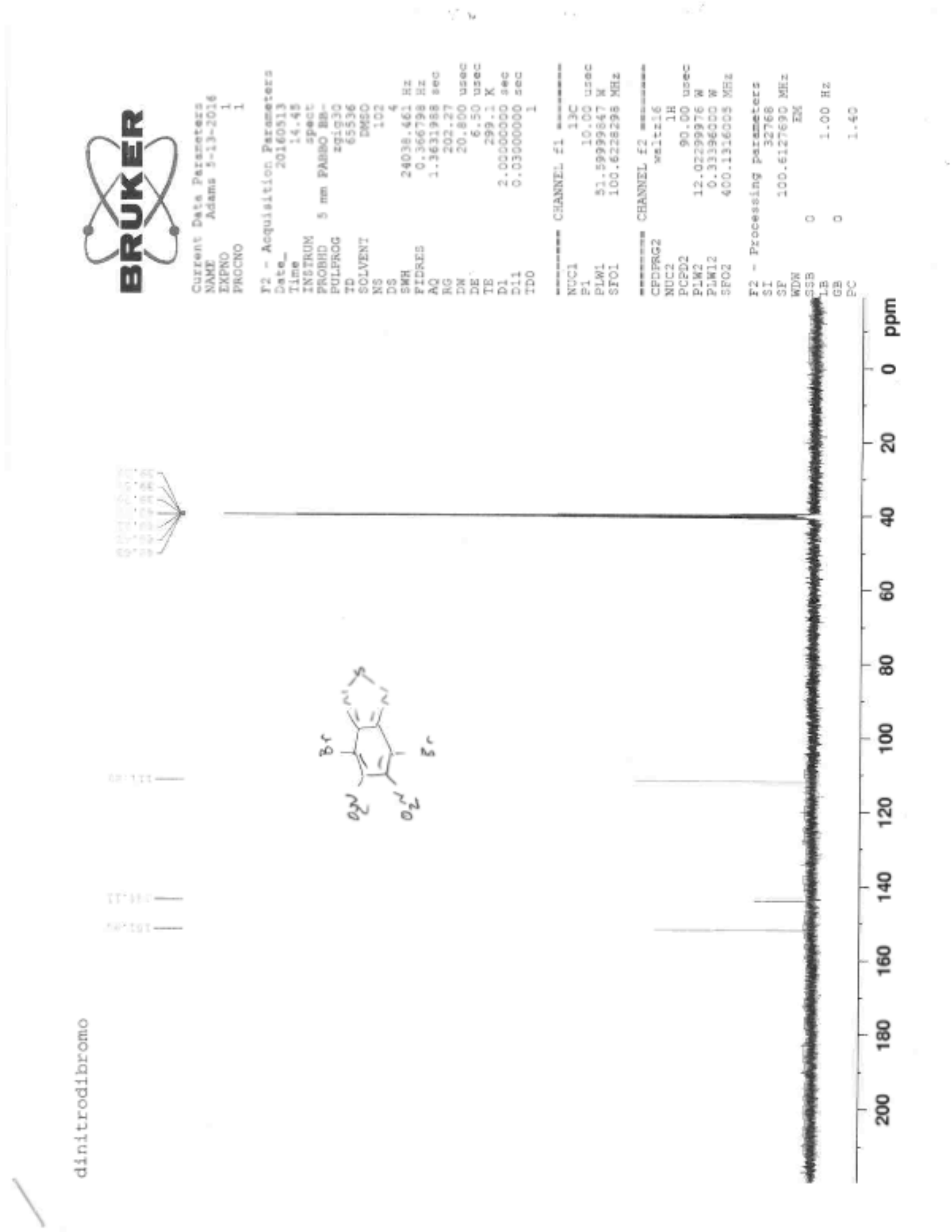

Figure S1. ^{13}C NMR of **3** in DMSO-d6 for comparison to **2** (below in Figure S2).

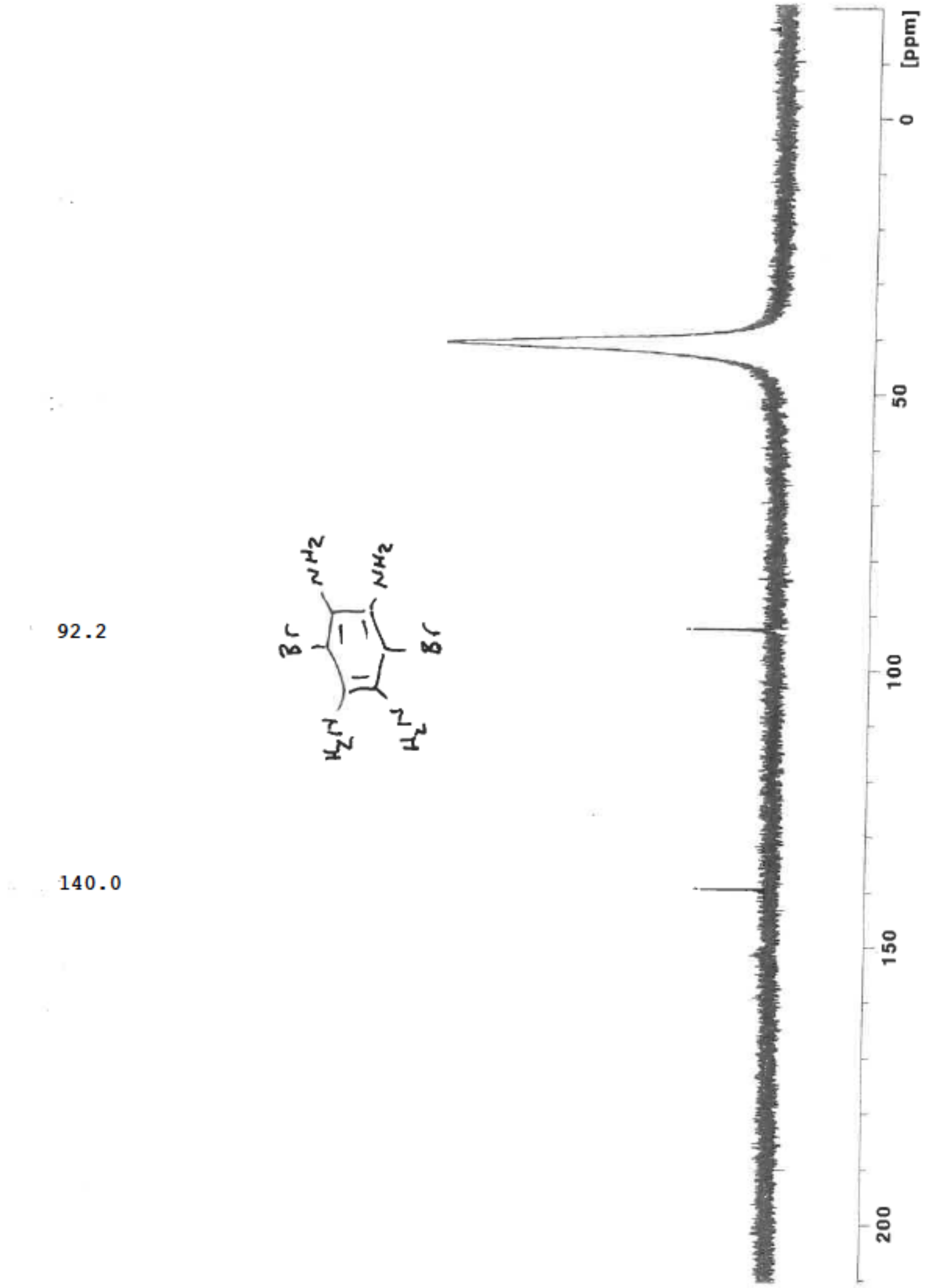

Figure S2. ^{13}C NMR of **2** in DMSO-d6.

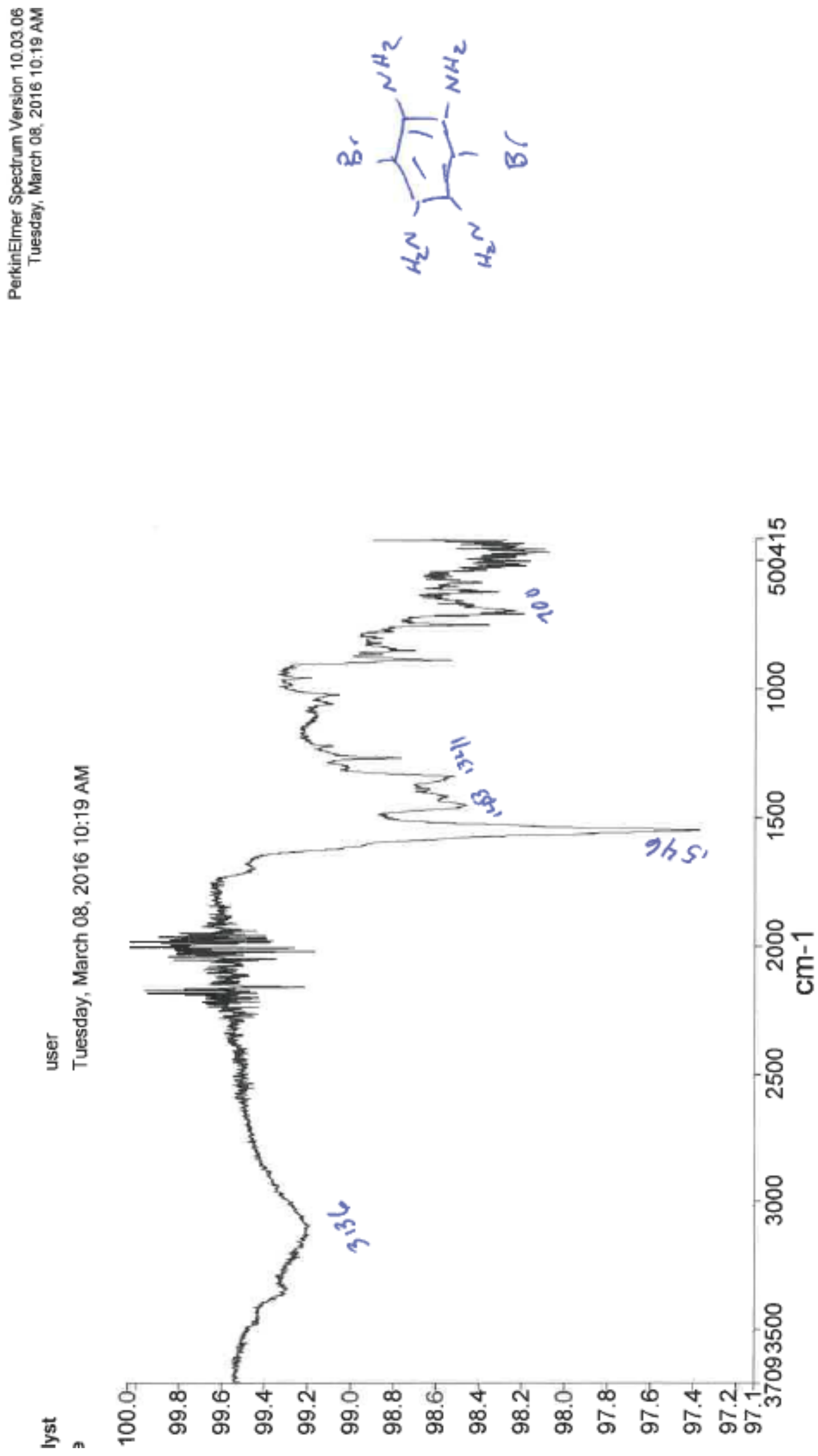

Figure S3. IR of **2.**

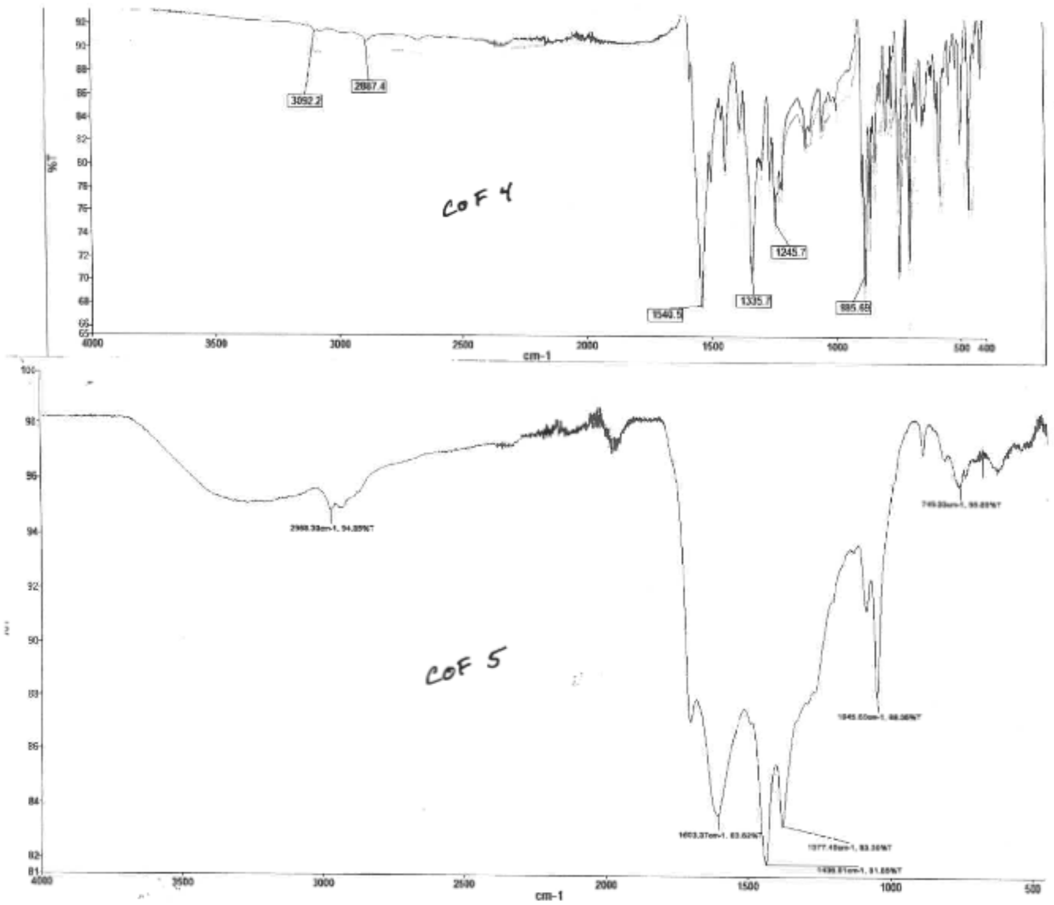

Figure S4. IR of COF **4** (top) & **5** (bottom).

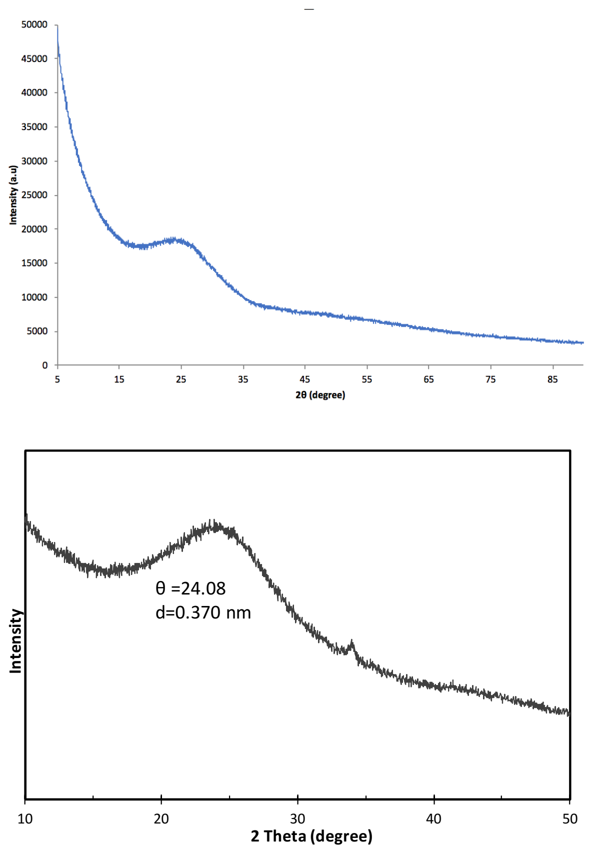

Figure S5. PXRD of COF **4** top and COF **5** bottom.

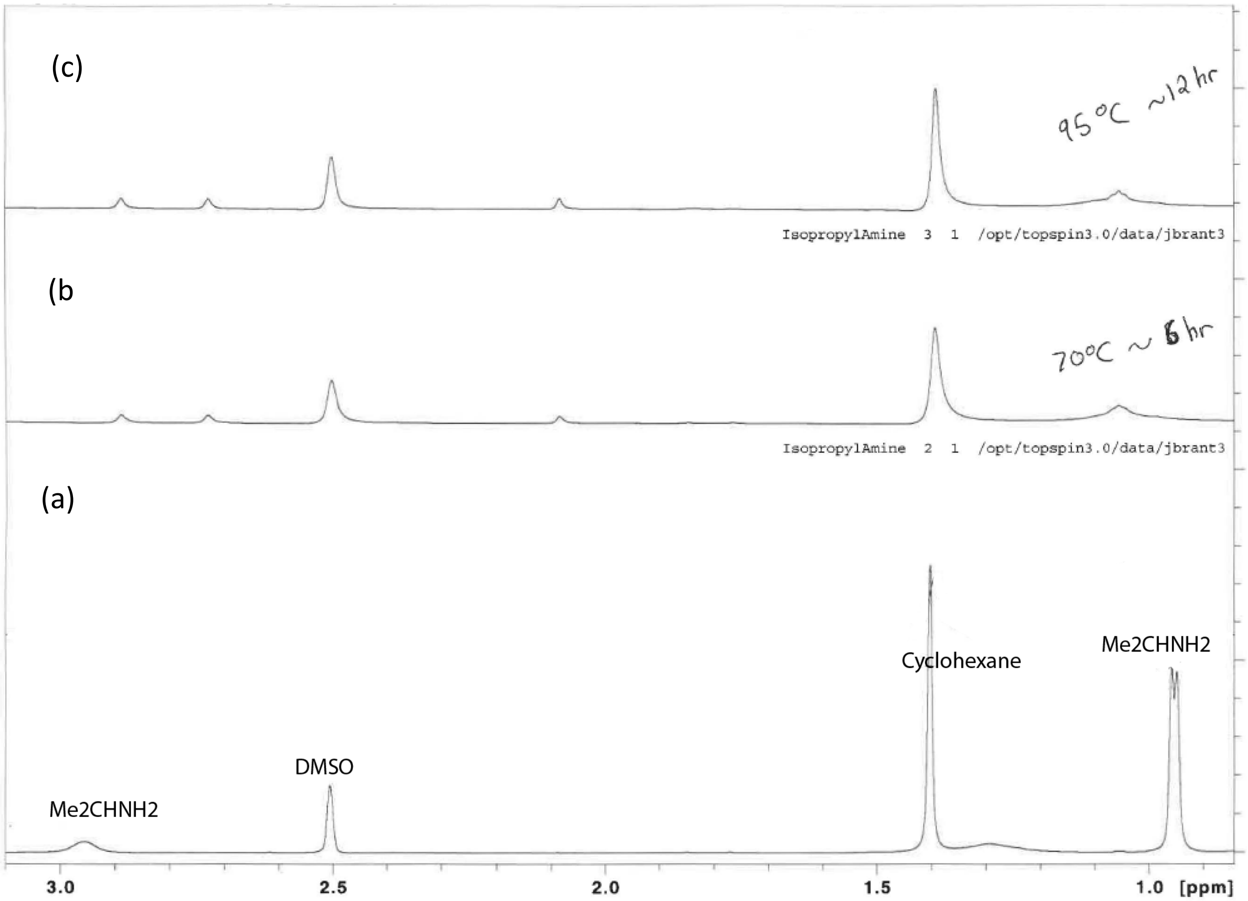

Figure S6. ^{1}H NMR experiment showing the incorporation of $iPrNH_2$ into COF **4**. A 20.5 mg sample COF **4** (equal to 0.154 mmol HKH and 0.154 mmol **2**) was reacted with isopropyl amine (18ul, 0.22 μmol). Spectrum (a) is time zero. Spectrum (b) is after 6 hours and (c) is 12 h in which all the CH_3 signals at 0.95 have disappeared. After filtration of the solid COF, the new peak at 1.05 disappears.

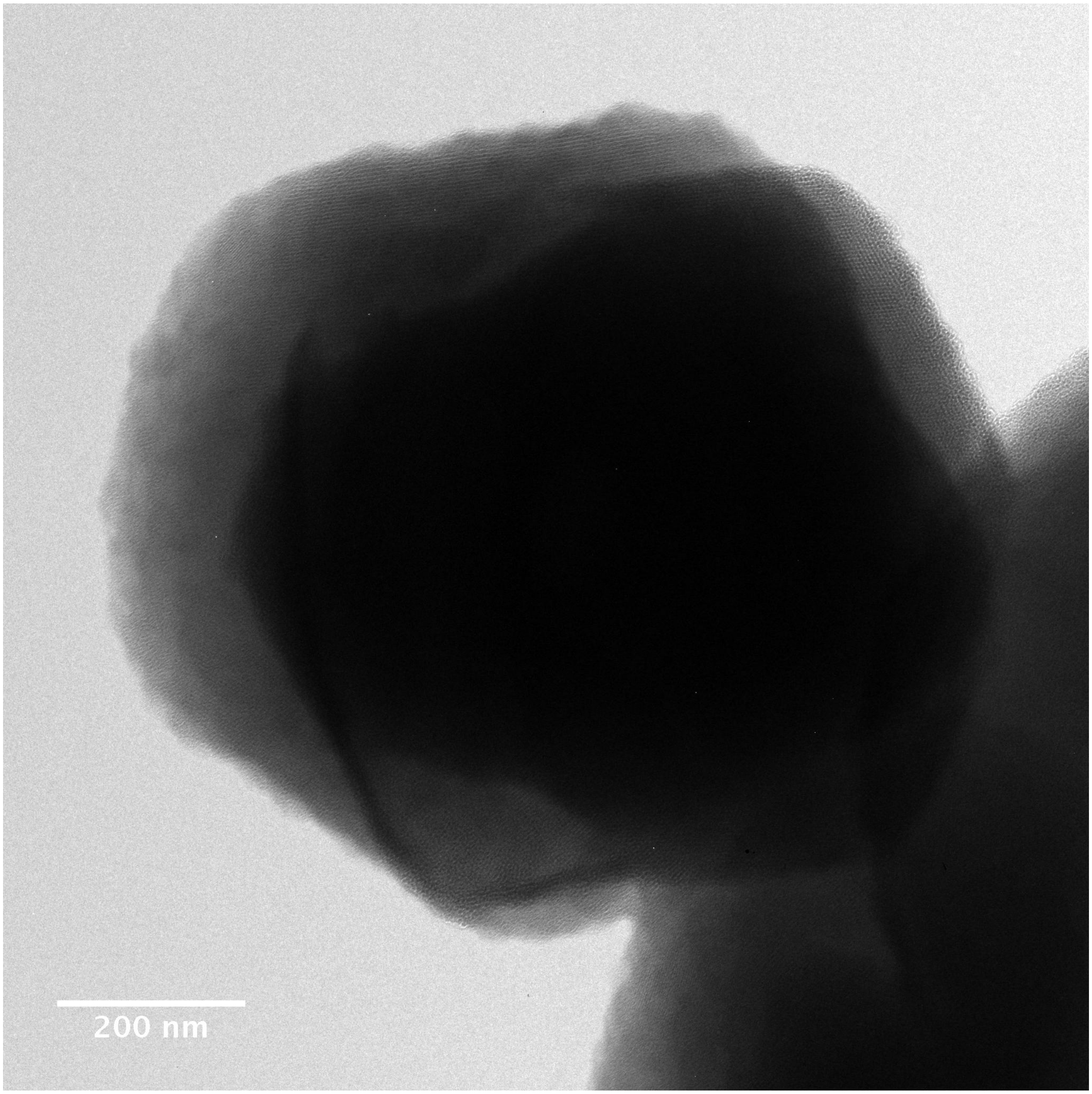

Figure S7. Enlarged TEM image of COF **4** from Figure 2 of manuscript

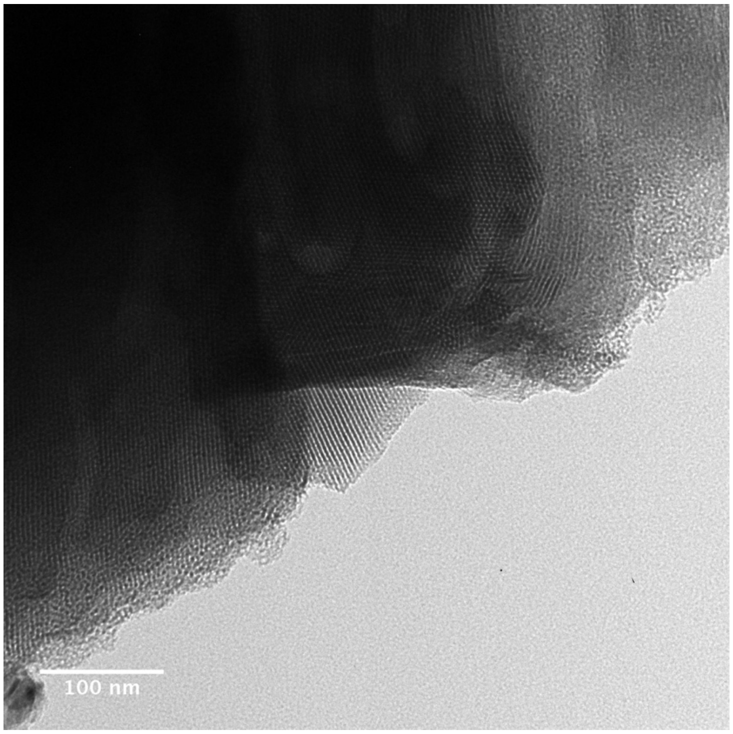

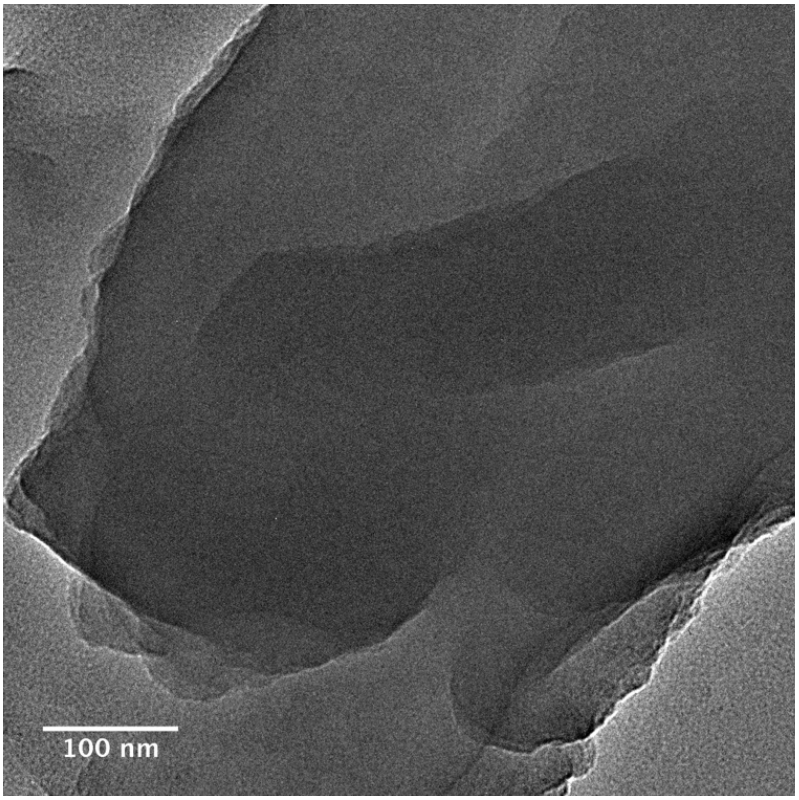

Figure S8. Enlarged TEM image of COF **4** from Figure 2 of manuscript (top); and TEM image of COF **4** made from tetraamine **2** synthesized by reduction of **3** using hydrogenation over $Pd(OH)_2/C$ in ethanol.

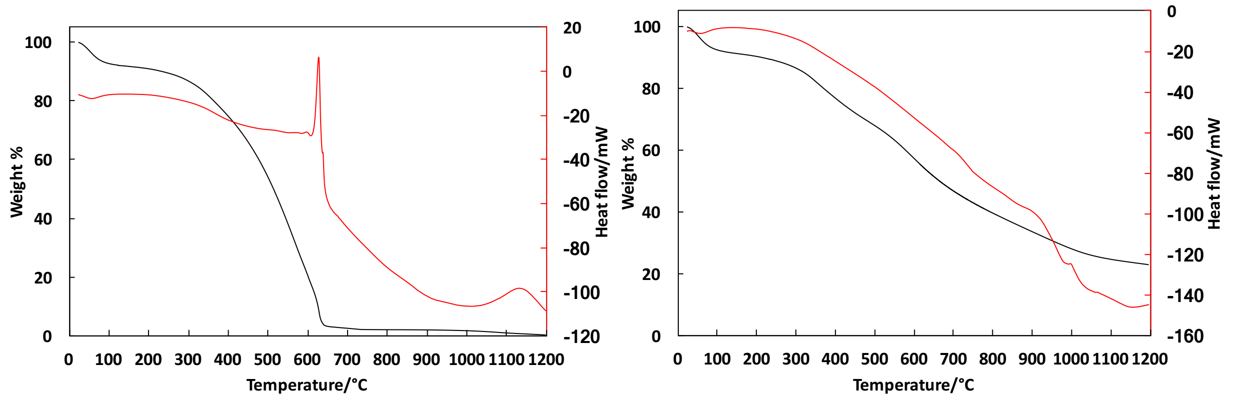

Figure S9. Thermogravimetric analysis and differential scanning calorimetry on COF **5** under (a) air and (b) argon**.** TGA/DSC analysis obtained from 25 °C to 1200 °C at a heating rate of 3 °C min^{-1}of the material under both argon and air shows a loss of mass at low temperatures indicating absorbed or adsorbed solvent or water also providing additional mass without carboxyl moieties. TGA/DSC curves under air showed the COF was completely oxidized at 600°C. TGA/DSC under argon atmosphere shows that the COF **5** is stable up to 400 °C with ~30% weight loss and further gradual weight loss past 900 °C. Decarboxylation maybe responsible for the initial weight loss.

Table 1. Radii and hydrated radii of R_4N^+ cations.

Cations	Ionic radii	Hydrated radii
NH_4^+	1.48 Å	3.31 Å
Me_4N^+	2.85 Å	3.47 Å
Et_4N^+	3.48 Å	4.00 Å
Bu_4N^+	4.37 Å	4.94 Å

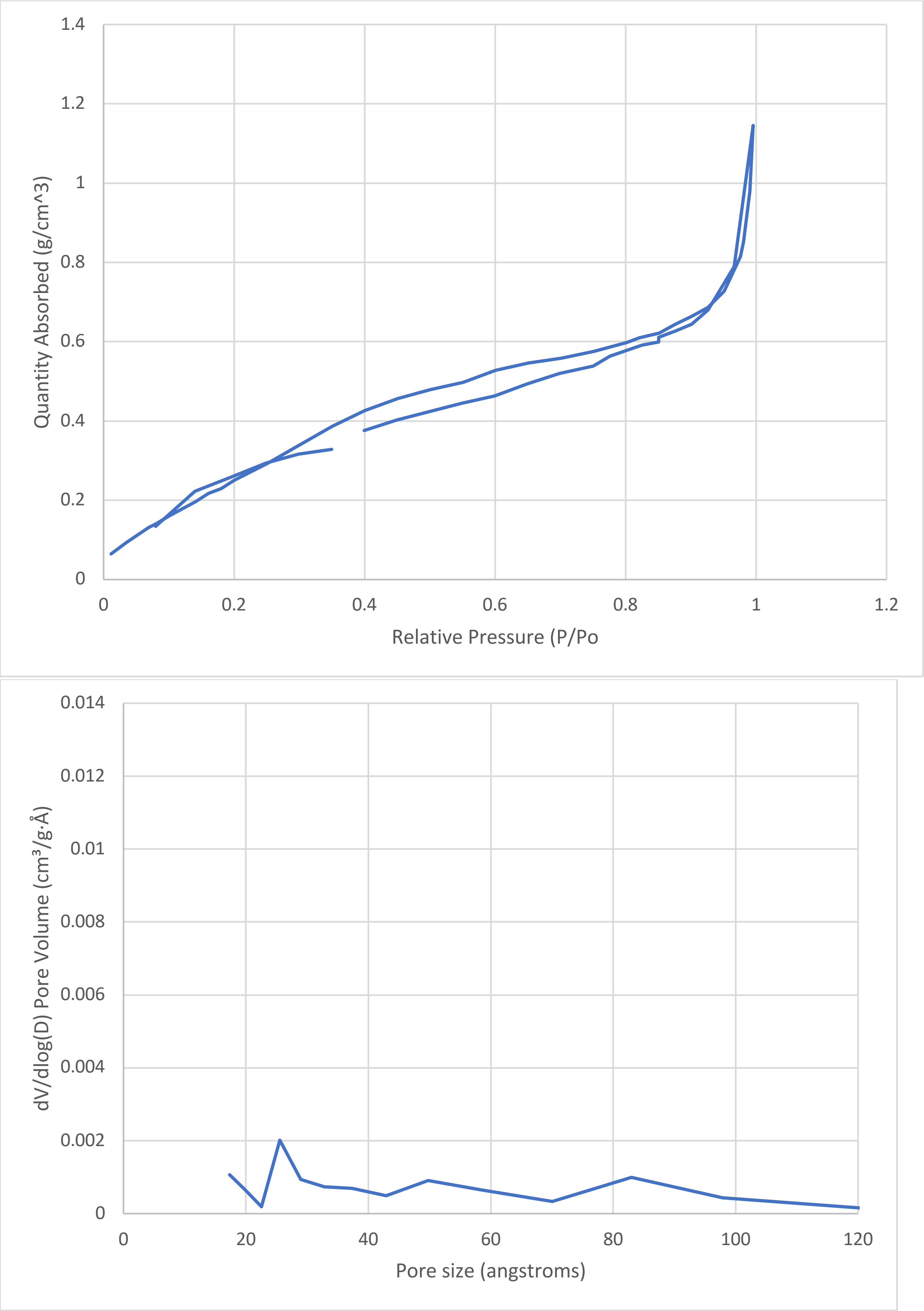

Figure S10. BET Surface area plot (top) and pore size distribution of COF **5.**
